# Sensorimotor mu rhythm during action observation changes across the lifespan independently from social cognitive processes

**DOI:** 10.1016/j.dcn.2019.100659

**Published:** 2019-05-17

**Authors:** Victoria E.A. Brunsdon, Elisabeth E.F. Bradford, Heather J. Ferguson

**Affiliations:** University of Kent, United Kingdom

**Keywords:** EEG, Mu rhythm, Mirror system, Sensorimotor processes, Social cognition, Developmental trajectories

## Abstract

The observation of actions performed by another person activates parts of the brain as if the observer were performing that action, referred to as the ‘mirror system’. Very little is currently known about the developmental trajectory of the mirror system and related social cognitive processes. This experimental study sought to explore the modulation of the sensorimotor mu rhythm during action observation using EEG measures, and how these may relate to social cognitive abilities across the lifespan, from late childhood through to old age. Three-hundred and one participants aged 10- to 86-years-old completed an action observation EEG task and three additional explicit measures of social cognition. As predicted, findings show enhanced sensorimotor alpha and beta desynchronization during hand action observation as compared to static hand observation. Overall, our findings indicate that the reactivity of the sensorimotor mu rhythm to the observation of others’ actions increases throughout the lifespan, independently from social cognitive processes.

## 1 Introduction

The observation of actions performed by someone else can activate neurons in the sensorimotor cortex, and this apparent mirroring of observed actions in the brain has led to the term ‘mirror neurons’. Mirror neurons were first termed after single unit studies reported cells in the premotor cortex of macaque monkeys that discharged both when performing actions and when observing actions (Dipellegrino et al., 1992). There is abundant evidence for a mirror neuron system in humans (Fox et al., 2016; Gallese et al., 2004; Kilner and Lemon, 2013), wherein observed actions are integrated with the person’s motor repertoire to understand actions (Calvo-Merino et al., 2005). The mirror system is thought to be configured through sensorimotor learning, i.e., through the repeated co-occurrence between a sensory input and motor output (Catmur and Heyes, 2013; Catmur et al., 2007, 2008, 2009; Cook et al., 2014; Heyes, 2001). However, very little is known about how this mirror system develops across the lifespan as most studies in humans have focussed exclusively on infants, young adults, or those with autism spectrum disorder. Therefore, it is not known whether and how the mirror system changes over typical development, or whether a comparably functioning mirror system is present in older adults. We address this gap in the literature by exploring for the first time how the mirror system develops across the lifespan, from late childhood through to old age, and how social cognitive processes are related to the functioning of the mirror system.

Electroencephalography (EEG) methods have been used to assess the modulation of the sensorimotor mu rhythm during both action execution and observation as a proxy of the mirror system (Arnstein et al., 2011; Fox et al., 2016). The mu rhythm is an EEG oscillation between 8 and 13 Hz (Hari et al., 1997) recorded from central areas overlying the sensorimotor cortex. There has been recent debate regarding the distinction between the mu rhythm and alpha activity, since both are composed of the same frequency bands (Bowman et al., 2017; Fox et al., 2016; Hobson and Bishop, 2016). Mu and alpha have largely been distinguished based on their topography, with mu originating from central areas (overlying the sensorimotor cortex) and alpha originating from occipital areas (overlying the occipital lobe). Mu desynchronization studies have also considered beta oscillations from 13 to 35 Hz (Hobson and Bishop, 2017a,b), as the mu rhythm appears to consist of two spectral peaks at ˜10 Hz and ˜20 Hz (Hari, 2006). At rest, the sensorimotor cortex activity is synchronous, but during both action execution and observation the sensorimotor cortex activity becomes desynchronized, reflecting changes in cortical activity (Fox et al., 2016). Typically, EEG mu desynchronization studies compare mu power in a baseline condition (e.g., static hands (Puzzo et al., 2011, or kaleidoscope videos, Hobson and Bishop, 2017a, Hobson and Bishop, 2017b) with an experimental condition (e.g., performing and/or observing hand actions, Puzzo et al., 2011). A reduction in mu power over central regions in the experimental condition as compared to the baseline condition indicates that the mu rhythm has desynchronized to the performance/observation of actions, reflecting changes in the activation of the sensorimotor cortex.

Mu desynchronization studies have been expanded to investigate whether the mirror system is an important mechanism for social cognition, such as for imitation (e.g., Braadbaart et al., 2013), theory of mind (e.g., Pineda and Hecht, 2009), and empathy (e.g., Woodruff et al., 2011), as well as to investigate whether a ‘faulty’ mirror system underlies autism spectrum disorder (Oberman et al., 2005). However, there are a growing number of studies that dispute the role of the mirror system in understanding others’ actions and intentions (see Hickok, 2009, 2013). For example, individuals unable to execute actions due to congenital upper limb dysplasia were still able to understand and interpret those actions, undermining the view that the sensorimotor cortex mirrors observed actions to allow understanding and interpretation of others’ behaviour (Vannuscorps and Caramazza, 2016). There is abundant evidence of sensorimotor cortex activation to action observation, but the function of this observation-related sensorimotor activity is therefore unclear. More recently, Catmur et al. (2018) demonstrated that ‘counter-mirror’ sensorimotor training (associative training in which the observation of one action is paired with the performance of another action) significantly reduced action understanding, providing support for the role of the mirror system in action understanding. We extend this work by investigating the developmental relationship between the mirror system and higher-order social cognitive processes.

Thus far, the majority of mu desynchronization studies in healthy individuals have focussed on the emergence of mirror system activity in infancy (˜9 months old; Lepage and Theoret, 2006), or on averaged data from younger adults derived from student populations (e.g., 18–33 years old: Hobson and Bishop, 2016; 18–34 years old: Perry and Bentin, 2009; 21–41 years old: Muthukumaraswamy et al., 2004). To our knowledge, there have been no developmental studies of sensorimotor mu rhythm across the lifespan, with a paucity of research in both adolescence and older age, meaning that very little is known about its development beyond childhood. The limited research that has addressed this developmental trajectory has observed changes across the mirror system network in early childhood (Shaw et al., 2012); children aged 10 years old elicit comparable brain activation during action observation as adults (Biagi et al., 2016), which suggests that mirror system development may reach maturity by mid/late-childhood. However, adolescence is a period of substantial development of certain areas of the brain involved in social cognitive processes (Blakemore, 2008), thus it is possible that the mirror system continues to change beyond mid/late-childhood. Crucially, it remains unknown whether and how the mirror system continues to develop across adolescence and into adulthood (Kilner and Blakemore, 2007).

To our knowledge, there have been no sensorimotor mu rhythm studies investigating action observation in healthy aging. However, research has reported behavioral declines in related social abilities in older age, including theory of mind (Henry et al., 2013), action learning (Coats et al., 2013), and imitation (Maryott and Sekuler, 2009). Moreover, functional imaging has revealed that motor-related areas in the brain are susceptible to aging, leading to compensatory over-activation in the motor cortices during action execution tasks (Hutchinson et al., 2002; Riecker et al., 2006; Ward and Frackowiak, 2003). EEG studies that have investigated aging effects on action execution using the go/no-go paradigm have reported greater beta desynchronization for response suppression, movement preparation and execution in older adults (Schmiedt-Fehr et al., 2016), leading to the suggestion that additional brain networks are recruited in older age (Hong et al., 2016). The current study examines whether the reactivity of the sensorimotor mu rhythm during action observation shows a comparable increase with age.

The aim of the current study was to explore the functioning of the mirror system across the lifespan, from late childhood through to old age, to obtain a comprehensive picture its development. In addition, we investigated how behavioral changes in social cognitive processes map onto the functioning of the mirror system across the lifespan. As a proxy of mirror system functioning, we investigated the modulation of the sensorimotor mu rhythm to the observation of other’s hand actions. It was predicted that, across all ages, there would be greater sensorimotor mu and beta desynchronization during hand action observation compared to static hand observation, replicating previous findings (Puzzo et al., 2010). Moreover, in line with research that has shown increased beta desynchronization in older adults during action execution (Hutchinson et al., 2002; Riecker et al., 2006; Ward and Frackowiak, 2003), we expected to observe a greater action-static difference in mu/alpha and beta desynchronization for older adults compared to young adults. Importantly, we also explored whether increasing age and higher social cognitive processes, including empathy (Empathy Quotient; EQ), emotion recognition (Reading the Mind in the Eyes Task; RMET), and theory of mind (ToM; Strange Stories), are related to the functioning of the mirror system.

## 2 Method

### 2.1 Participants

In total, 354 participants completed the larger CogSoCoAGE study. The final CogSoCoAGE sample consisted of 350 participants, as two participants were excluded due to low IQ (<70), one participant was excluded due to being a non-native English speaker, and one participant’s data was lost due to computer failure. All participants were native English-speakers, had normal or corrected-to-normal vision, had no known neurological disorders, and had no mental health or autism spectrum disorder diagnoses. The participants' consent was obtained according to EU legislation, and the Ethical Committee of the School of Psychology, University of Kent, approved the study.

From the original sample, 14 participants did not complete the EEG task, 11 participants were excluded due to excessive noise on the EEG recordings, nine participants were excluded due to too few segments for the EEG analysis (less than two-thirds of segments remaining), three participants were excluded due to computer error on the EEG task, three participants were excluded due to outliers in the EEG data, one participant did not complete the Reading the Mind in the Eyes Task, one participant was excluded due to computer error on the Strange Stories, and seven participants did not complete the Empathy Quotient. Thus, the final sample consisted of 301 participants in total, aged 10–86 years old (207 females, 94 males).

### 2.2 Measures

#### 2.2.1 Action observation EEG task

This task was adapted from a previous study (Puzzo et al., 2011) and was used to measure sensorimotor mu/alpha and beta desynchronization during hand action observation compared to static hand observation as a proxy of the human mirror system. First, participants performed a resting EEG for 2 min, which involved fixating on a central cross on a grey screen. After a self-directed break, participants performed the action observation EEG task that contained 60 experimental trials. Stimuli consisted of seven different video clips depicting a static hand or various hand actions: cutting a piece of paper with scissors, ringing a bell, dialling a number on a mobile phone, clicking fingers, locking a door with keys, and crumpling a piece of paper. Trials consisted of a 1000 ms fixation cross, then a 3000 ms video clip, ending with a 1000–3000 ms blank screen (the inter-trial interval was variable to prevent expectancy effects on mu rhythm). Each of the six hand action video clips was shown five times with a total of 30 hand action trials. The static hand video clip was shown 30 times with a total of 30 static hand trials. Trials were presented in a randomised order. There was a break halfway through the task, the duration of which was directed by the participant.

#### 2.2.2 Social cognition tasks

Three explicit measures were used to examine higher social cognitive processes, including emotion recognition, theory of mind and empathy (see supplementary materials for more detail [S1]).

A computerised version of the Reading the Mind in the Eyes Task (RMET) was completed using 28 items for the child version (10–15 years old) or 36 items for the adult version (Baron-Cohen et al., 2001). Participants’ response accuracy was recorded (*M* = 73.75%, range = 44.44–94.44%).

A computerised version of the Strange Stories was completed, in which participants verbally responded to questions about eight theory of mind, eight physical and eight nature stories (White et al., 2009). A ToM score was calculated from the theory of mind stories (*M* = 13.51, range = 2–16) and a non-ToM control score was calculated from the physical stories (*M* = 13.74, range = 4–16).

The parent-report (10–15 years old; Auyeung et al., 2012) or the self-report (16+ years old; Baron-Cohen and Wheelwright, 2004) version of the Empathy Quotient (EQ) was completed. The questionnaires were scored to gain a total empathy score (maximum = 80) with a low score indicating low levels of empathy and a high score indicating high levels of empathy (*M* = 46.24, range = 7–76).

### 2.3 Procedure

Participants (or their parents if aged 10–15 years old) completed the EQ (plus additional questionnaires) before attending testing sessions. Participants completed one or two visits to the university as part of a larger study, which lasted approximately 5 h in total. The RMET and Strange Stories were included in a larger task battery with tasks administered in a counterbalanced order. The action observation EEG task was always completed at the end of the testing session. The Acticap was first applied and set up for recording. Participants then completed the action observation EEG task while EEG activity was recorded.

### 2.4 EEG recording and analysis

Electroencephalographic (EEG) activity was recorded during the action observation EEG task from 30 active electrodes using a Brain Vision Quickamp amplifier system with an ActiCap cap referenced to FCz. Vertical electro-oculogram (VEOG) activity was recorded from one extra electrode (below right eye), and horizontal electro-oculogram (HEOG) activity was recorded from one extra electrode (to the left of the left eye). EEG and EOG recordings were sampled at 1000 Hz, and electrode impedance was kept below 10kΩ.

Prior to segmentation, a vertical ocular calculation was applied (1*Fp2+(-1*VEOG)). All data were re-referenced to a common average reference. EEG and EOG activity were band-pass filtered (0.1–70 Hz, notch filter at 50 Hz). Data were visually inspected for noisy sections or channels, and for other general artifacts. EEG activity containing blinks was corrected using a semi-automatic ocular ICA correction approach (Brain Vision Analyzer 2.1). An average of 3 ICA components were removed per individual dataset.

The 2-minute resting EEG data was then cut in to 2 s epochs (starting 0–2000 ms). Semi-automatic artifact detection software (Brain Vision Analyzer 2.1) was run, to identify and discard segments with non-ocular artifacts (drifts, channel blockings, EEG activity exceeding ± 50μV). A fast-fourier transformation, with 10% Hanning window, was then applied to each segment. The average alpha (8–13 Hz) and beta power (13–35 Hz) at rest was then calculated across all artifact-free segments for each electrode of interest. There was an overall data loss of 5.24% for the resting EEG, with an average of 57 (out of 60) baseline segments retained per participant.

The action observation EEG task trial data segments (hand action and static hand) were cut into 2 s epochs (500–2500 ms from stimulus onset). Semi-automatic artifact detection software (Brain Vision Analyzer 2.1) was run, to identify and discard segments with non-ocular artifacts (drifts, channel blockings, EEG activity exceeding ± 50μV). A fast-fourier transformation, with 10% Hanning window, was then applied to each segment, and the signal was averaged for each condition and electrode. There was an overall data loss of 6.02% for the hand action trials and 6.49% for the static hand trials, with an average of 28 (out of 30) trial segments retained per participant.

The average mu/alpha (8–13 Hz) and beta (13–35 Hz) power for each condition was calculated for the electrodes of interest over the central (C3, Cz, C4) and occipital electrodes (O1, Oz, O2). This allowed us to test whether changes in mu and beta desynchronization over central sites were distinct from alpha and beta desynchronization over occipital sites (Hobson and Bishop, 2017a,b). A measure of the percentage change in power for each condition (test: hand action or static hand) and the resting EEG as a reference period (reference) was calculated for each electrode of interest for both alpha and beta bands, using the formula: (reference-test/reference) × 100 (Puzzo et al., 2011). Any outlier data points were excluded for both alpha and beta desynchronization values (+/−3SD from the mean). Data from electrodes C3, Cz and C4 were averaged for the central electrode site, and data from electrodes O1, Oz and O2 were averaged for the occipital electrode site. Positive values indicate alpha and beta desynchronization and negative values indicate alpha and beta synchronization.

### 2.5 Statistical analysis

All statistical analyses were conducted in R version 3.5.0. The data and code are available at osf.io/z2ndf. To investigate alpha and beta desynchronization during the action observation EEG task, two 2 × 2 repeated measures ANOVAs were performed, with condition (hand action, static hand) and electrode site (central, occipital) as within-subject variables, and alpha and beta desynchronization values as the dependent variables. Post-hoc paired-samples *t*-tests (two-tailed) were conducted to investigate the source of significant interactions.

To investigate relationships with age, a series of regression models tested linear and curvilinear relationships between alpha/beta desynchronization to hand actions versus static hands during the action observation EEG task and age, and for each social cognition measure and age. The first series of models specified the outcome variable as the action-static difference in power across the central electrodes in the alpha or beta band and the predictor variable as age using linear, quadratic, cubic, or quartic terms. The best fitting model was deduced by comparing the simpler model against the more complex model using an ANOVA (i.e., linear vs. quadratic, quadratic vs. cubic, cubic vs. quartic; see supplementary materials [S2]). If the p-value was greater than 0.05, then the simpler model was selected as the best fitting model. The best fitting model was then re-run with the addition of the action-static difference in power across the occipital electrodes as a covariate. If a curvilinear relationship with age was determined, follow-up linear regressions were performed by subsetting the data into appropriate age bands to further describe any increases or decreases across certain periods of age. This sequence of model fitting was then repeated separately for each of the following outcome variables: (1) percentage correct for RMET, (2) ToM score and (3) physical score for Strange Stories, and (4) total score in the EQ. If a curvilinear model provided the best fit, then the curve was examined with a series of linear regressions to further describe the relationship with age. Finally, two multiple regression models were conducted to examine whether age and the social cognitive measures are related to the alpha and beta desynchronization during the action observation EEG task. These models specified alpha/beta desynchronization across the central electrodes as the outcome variables, and age, RMET, ToM and physical score in Strange Stories, EQ total as predictor variables, and alpha/beta desynchronization across the occipital electrodes as a covariate.

## 3 Results

### 3.1 Action observation

**Fig. 1:**
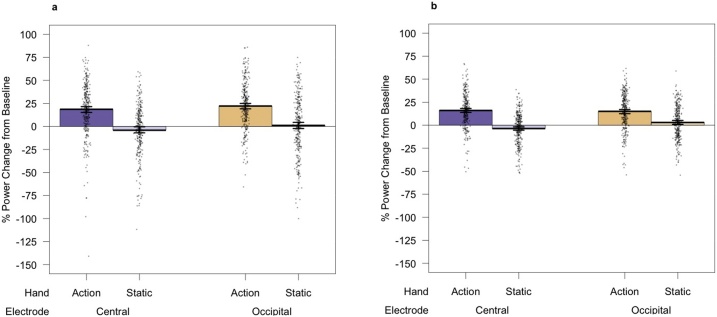
Mean percentage change in power from baseline for static hand observation and hand action observation over the central and occipital electrodes in alpha (**a**) and beta (**b**) bands. The bold horizontal line indicates the group mean and the bars indicate the 95% confidence intervals. The points show the raw data. Positive values indicate alpha and beta desynchronization and negative values indicate alpha and beta synchronization.

Fig. 1 shows the mean percentage change in power from baseline during static hand observation and hand action observation over the central and occipital electrodes for both alpha and beta bands.

A 2 (condition) x 2 (electrode) repeated measures ANOVA revealed a significant main effect of condition in both the alpha (*F* (1, 300) = 335.60, *p* < 0.001, η^2^ = .528) and beta band (*F* (1, 300) = 439.40, *p* < 0.001, η^2^ = .594), showing significantly greater desynchronization during hand action observation (alpha *M* = 20.38%; beta *M* = 15.55%) compared to static hand observation (alpha *M* = −1.52%; beta *M* = −0.30%). A significant main effect of electrode site was found for both the alpha (*F* (1, 300) = 9.75, *p* = 0.002, η^2^ = .032), and beta band (*F* (1, 300) = 7.92, *p* = 0.005, η^2^ = .026), indicating greater power over the occipital electrodes (alpha *M* = 11.63%; beta *M* = 9.03%) compared to the central electrodes (alpha *M* = 7.23%; beta *M* = 6.21%). There was also a significant 2-way interaction between condition and electrode in the beta band (*F* (1, 300) = 79.77, *p* < 0.001, η^2^ = .210). To examine this 2-way interaction, follow up analyses compared the magnitude of the action-static difference in power across the central versus occipital electrode sites. The action-static difference in power was significantly greater over the central site (*M* = 19.65%) compared to the occipital site (*M* = 12.05%) for the beta band (*t* (300) = 7.60, *p* < 0.001).

### 3.2 Relationship with age and social cognition

To more closely examine changes in the modulation of sensorimotor mu desynchronization to action observation over the lifespan, a series of regression models tested linear and curvilinear relationships between alpha/beta desynchronization and age (see supplementary materials [S2] for model comparisons). This was repeated for each social cognition measure to further examine social cognition across the lifespan. Finally, a multiple regression was conducted to investigate the relationship between alpha/beta desynchronization, age, and social cognition, whilst accounting for activity over the occipital electrodes.

**Fig. 2:**
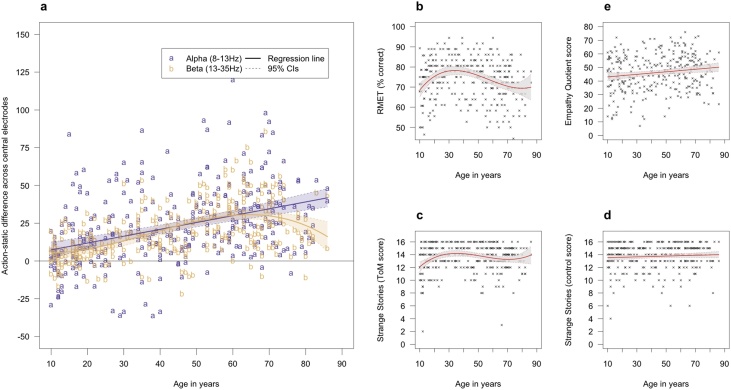
Relationship between age (in years) and a) the mean percentage change in alpha and beta power in the central electrodes from static hand observation to hand action observation in the action observation EEG task, b) percentage correct in Reading the Mind in the Eyes Task (RMET), c) Empathy Quotient (EQ) total score, d) Strange Stories Theory of Mind (ToM) score, and e) Strange Stories control score. The bold line indicates the best-fitting regression line and the dashed line indicates the 95% confidence intervals (CIs).

To examine changes in alpha/beta desynchronization, a difference score was calculated for each participant by subtracting the percentage change in power for the static hand condition from the percentage change in power for the action hand condition in the action observation task, separately for alpha and beta power bands, across the central electrodes. Mean difference scores per participant are displayed in Fig. 2a, showing age as a continuous variable.

A linear model provided the best fit of the data for the relationship between the action-static difference in alpha power and age (alpha: R^2^ = 0.14, *F*(1, 299) = 48.93, *p* *<* 0.001) and a cubic model provided the best fit of the data for the relationship between the action-static difference in beta power and age (R^2^ = 0.29, *F*(3, 297) = 40.44, *p* *<* 0.001). Age significantly explained the variance in the action-static difference over the central electrodes in both the alpha and beta power bands (alpha: *β* = 0.37, *p* < 0.001; beta: *β* = 0.79, *p* < 0.001; *β ^2^* = −0.17, *p* = 0.005; *β ^3^* − 0.18, *p* = 0.006). In the alpha band, desynchronization to hand actions versus static hands increased linearly from 10 to 86 years of age. To further describe the curvilinear relationship with age in the beta band, linear regressions on certain age periods were conducted; *β* estimates and *p*-values are presented to indicate significant relationships for each age period. In the beta band, desynchronization to hand actions versus static hands did not change between 10 to 17 years old (*β* = 0.13, *p* = 0.372), but increased from 18 to 60 years of age (*β* = 0.44, *p* < 0.001), then decreased from age 60 onwards (*β* = −0.27, *p* = 0.013). These relationships remained when accounting for the action-static difference over the occipital electrodes (alpha: R^2^ = 0.35, *F*(2, 298) = 83.32, *p* *<* 0.001; beta: R^2^ = 0.48, *F*(4, 296) = 68.52, *p* *<* 0.001).

A cubic model provided the best fit of the relationship between RMET and age (R^2^ = 0.09, *F*(3, 297) = 9.54, *p* < 0.001). Age was significantly associated with RMET scores (all beta coefficient *p*s < 0.012), indicating an increase in RMET scores from 10 to 35 years of age (*β* = 0.34, *p* < 0.001), a decrease from 35 to 70 years old (*β* = −0.22, *p* = 0.009), and a plateau from 70 years old (*β* = −0.13, *p* =  0.395; Fig. 2b).

A linear model provided the best fit of the relationship between EQ and age (R^2^ = 0.02, *F*(1, 299) = 7.07, *p* = 0.008). Age was significantly associated with EQ (*β* = 0.15, *p* = 0.008) indicating an increase in levels of empathy with increasing age (Fig. 2c).

A cubic model provided the best fit of the relationship between Strange Stories ToM scores and age (cubic R^2^ = 0.05, *F*(3, 297) = 5.59, *p* < 0.001). Age was significantly associated with Strange Stories ToM scores (all beta coefficient *p*s < 0.002), indicating an initial increase in ToM scores from 10 years to 30 years of age (*β* = 0.26, *p* = 0.008), a decrease from 30 to 70 years old (*β* = −0.17, *p* = 0.028), and a plateau from 70 years of age onwards (*β* = −0.13, *p* = 0.395; Fig. 2d). Strange Stories control scores were not significantly associated with age (*β* = 0.07, *p* = 0.257; linear R^2^ = 0.004, *p* = 0.257; Fig. 2e).

Crucially, to explore the relationship between sensorimotor activity, age, and social cognition, a multiple linear regression was calculated to predict the action-static difference in alpha/beta desynchronization over the central electrodes based on age, RMET scores, Strange Stories ToM and control scores, and EQ score, accounting for the action-static difference over the occipital electrodes. The regression model was significant for both alpha (R^2^ = 0.37, *F*(6, 294) = 28.39, *p* < 0.001) and beta power (R^2^ = 0.47, *F*(6, 294) = 44.04, *p* < 0.001)^1^ . This analysis revealed that the action-static difference over the central electrodes was significantly predicted by the action-static difference over the occipital electrodes (alpha *β* = 0.49; beta *β* = 0.48), age (alpha *β* = 0.23; beta *β* = 0.38), and Strange Stories ToM score for beta power only (beta *β* = 0.10). None of the other predictors were significant (all *p*s > 0.211).

## 4 Discussion

The present study is the first to explore sensorimotor mu rhythm during action observation from late childhood through to old age. Three-hundred and one individuals aged 10- to 86-years-old observed short video clips depicting hand actions or a static hand, and mu/alpha (8–13 Hz) and beta (13–35 Hz) desynchronization were used as an EEG marker of mirror system activity across the sensorimotor cortex. Results revealed greater alpha and beta desynchronization across the sensorimotor cortex during hand action observation compared to static hand observation, in support of our predictions.

Importantly, our study is the first to explore the developmental trajectory of the mirror system from 10 years old through to 86 years old in a sizable sample. Analyses revealed a greater percentage change in the alpha power band over the central electrodes to hand action observation from 10 to 86 years old. In contrast, the percentage change in beta power over the central electrodes to hand action observation did not change through adolescence, but increased from 18 to 60 years old, then decreased in older age. These differential patterns over age for sensorimotor alpha and beta rhythms to observing actions suggest that these rhythms have distinct developmental trajectories. These distinct developmental trajectories likely reflect the dissociable, but complementary, processes underlying the two rhythms, with the alpha rhythm suggested to be related more to sensory processing and beta rhythm related more to motor processing (Ritter et al., 2009). These patterns therefore highlight the importance of measuring both alpha and beta power bands in EEG studies of action observation.

These age-related changes in the sensorimotor mu rhythm during action observation are a novel finding; the few existing studies that have examined aging and action execution/observation have generally compared a dichotomous sample of younger versus older adults (Hutchinson et al., 2002; Riecker et al., 2006; Schmiedt-Fehr et al., 2016), which does not allow the investigation of developmental trajectories from adolescence and through middle age. The overall action-static desynchronization effect found here shows that a functioning mirror system is present in late childhood and adolescence. However, in line with studies showing continued development of the ‘social brain’ during adolescence (Blakemore, 2008), our results reveal that the mirror system has not reached full maturity by adolescence; sensorimotor alpha desynchronization increased between adolescence and into adulthood, and sensorimotor beta rhythm remained the same during adolescence but increased into adulthood.

Crucially, our findings also suggest that the reactivity of the sensorimotor mu rhythm to action observation continues to change beyond adolescence, throughout adulthood and into older age. This enhanced sensorimotor alpha/beta rhythm into older age parallels that seen in previous research that has found over-activation of motor areas during action execution in older adults (Heinrichs-Graham et al., 2018; Rossiter et al., 2014; Schmiedt-Fehr et al., 2016; Vallesi and Stuss, 2010). We have provided novel evidence that this change emerges incrementally *throughout* adulthood, and is not tied specifically to the onset of old age (typically considered 65 years old plus). This related research interprets the increased activation in older age either as a compensatory aging mechanism, or as detrimental in nature (Ward, 2006). The compensatory account proposes that advancing age leads to increasing compensatory activity to maintain task performance, whereas the detrimental account proposes that advancing age leads to greater activity that causes poorer task performance. Our findings suggest that while sensorimotor mu desynchronization during action observation clearly increases through adulthood and into older age in the alpha band, activity in the beta band actually decreases from 60 years onwards. As such, the current data only provides partial support for these existing accounts, and suggests that any compensatory or detrimental activity reaches a peak around the onset of old age. It is also interesting to note that the static or declining mu desynchronization observed here in in older age would have been occluded in a group comparison design (e.g., 19–26-year-olds vs. 55–71-year-olds; Schmiedt-Fehr et al., 2016), or in studies that only test a linear relationship between beta desynchronization and age (Ritter et al., 2009).

An interesting alternative to these existing compensatory/detrimental accounts is that the increasing sensorimotor mu desynchronization across adulthood to action observation reflects *enhanced* specialization of the mirror system. This novel proposal is supported by previous research showing effects of expertise, where motor areas show greater activity while observing an action that is part of the observer’s motor repertoire (Calvo-Merino et al., 2006). Moreover, mu suppression has been shown to increase after active experience with actions, indicating that action expertise modulates the sensorimotor mu rhythm (Cannon et al., 2014; Marshall et al., 2009; Quandt et al., 2011). It is therefore possible that the increasing sensorimotor activation seen across adulthood might reflect individuals’ increasing experience/expertise with the observed motor actions. This possibility is particularly relevant in the current study since the observed videos depicted everyday hand actions (e.g. unlocking a door, dialling on a phone), meaning that participants’ experience with those actions was likely to increase with age due to more frequent encounters in everyday life. An expertise account would therefore be compatible with the continuously increasing effect seen across adulthood in the current study. Crucially, this account would suggest that the human brain continues to develop and specialize not just through adolescence and young adulthood (as previously documented by Blakemore, 2015), but well into middle age.

This study also explored the developmental trajectories of higher social cognitive processes. Participants completed three additional measures purported to assess complex emotion understanding, theory of mind ability and empathic capacity. Complex emotion understanding (as measured by the RMET) improved from adolescence through to adulthood with a peak at 35 years old, and a decline through middle age to 70 years old, and no change in older age. Empathy capacity (as measured by the EQ) showed a linear increase from adolescence into old age. Finally, theory of mind ability (as measured by the Strange Stories) showed an improvement from adolescence through to adulthood with a peak at 30 years old, a decline to 70 years old, and no further change in old age. In contrast, the control stories from the Strange Stories did not show a relationship with age, suggesting that age effects in this task are specific to social inferences and do not simply reflect a general decline in memory. Taken together, our social cognitive findings support research that has reported behavioral declines in older age in the understanding of complex emotions and mental states (Henry et al., 2013) and contrasts with research that has reported no age-related differences in empathy capacity (Beadle et al., 2012; Grühn et al., 2008). Therefore, this study adds to the literature as we demonstrate distinct developmental trajectories of different social cognitive processes that is occluded in past research that has used group comparison designs.

Importantly, we also explored whether increasing age and these higher social cognitive processes are related to the functioning of the mirror system. Increasing alpha desynchronization to action observation was related to increasing age, but was not related to any measure of social cognition, after controlling for desynchronization over the occipital cortex. Increasing beta desynchronization over the sensorimotor cortex to action observation was related to increasing age and theory of mind ability, after controlling for desynchronization over the occipital cortex. This indicates that there is an age-related change in alpha desynchronization that does not map onto a behavioral change in the social cognitive components tested here. In addition, this finding suggests that sensorimotor processes and social cognitive processes may be underpinned by distinct neural mechanisms, each with different developmental trajectories. This suggestion is consistent with a meta-analysis of more than 200 fMRI studies of the mirror and mentalizing systems (Van Overwalle and Baetens, 2009). This meta-analysis indicated that the mirror and mentalizing systems are both involved in the processing of sensory or verbal information about other people. However, the mirror and the mentalizing systems are rarely concurrently activated, with the mirror system activated by the observation of moving body parts when no active inferential processing is required and the mentalizing system activated when this input is not available (Brass et al., 2007; Van Overwalle and Baetens, 2009). In line with the conclusions of Brass et al. (2007), the distinction between mirror processes and social cognitive processes in the current study may be due to the degree of inferential processing needed to understand the actions, i.e., the familiar actions used here would be automatically mapped on to the person’s motor repertoire to understand the actions with little to no input from the mentalizing system, whereas the mirror system may be dependent on the mentalizing system when inferring the purpose of an action is more difficult, such as for understanding unfamiliar hand actions. One challenge for future work is to identify the degree of inferential processing needed to understand different types of actions, particularly from an aging perspective. For example, we find that mentalizing abilities decrease throughout adulthood, and as such, we would predict increased difficulties with age in action understanding for actions with greater levels of inferential processing (e.g., unfamiliar versus familiar actions).

The finding that theory of mind ability predicted beta desynchronization, but not alpha desynchronization, has interesting implications for the specificity of the relationship between mirror system development and social cognitive skills. Though we acknowledge that the effect size for the relationship is small even in the beta rhythm, we consider how this difference might relate to the different underlying processes that each rhythm is likely to reflect. Specifically, it has been suggested that beta desynchronization to the observation of an action reflects the activity of the motor cortex that guides motor preparation and selection, and supports the understanding of complex actions (Ritter et al., 2009). Therefore, a better understanding of other people (i.e., theory of mind) may be related to better understanding of goal-directed actions of other people, resulting in greater activity of the motor cortex during action observation. This is a tentative link that requires further investigation as we note that the current study mapped behavioral changes in a small set of general social cognitive processes using diverse measures and paradigms onto putative EEG markers of the mirror system. Future research should investigate how more specific motor skills map onto the functioning of the mirror system across the lifespan, preferably using a range of tasks that assess different components of social cognition, and elicit a behavioral response alongside the EEG measures. Some obvious candidates are imitation and grasp responses (Kumar et al., 2013), as both have been shown to modulate activity in the mirror system, which can be mapped onto changes at a behavioral level. We note that neither of these capacities have been explored in a lifespan context, meaning that significant open questions remain regarding whether and how changes in the mu desynchronization might predict decreasing motor control with advancing age (Seidler et al., 2011) or in clinical movement disorders (e.g. Parkinson’s disease, Caligiore et al., 2017).

In this paper we interpret our findings with the view that sensorimotor mu desynchronization to action observation reflects the activation of the mirror system. However, there is a debate regarding the extent to which mu desynchronization reflects mirror system activity (see Bowman et al., 2017; Hobson and Bishop, 2017a, b). For example, an alternative interpretation suggests that the central mu rhythm instead indexes somatosensory features of an action, rather than the motor features of an action (Coll et al., 2015; Cook et al., 2014), reflecting sensory processing rather than motor mirroring (Coll et al., 2017). Hobson and Bishop (2017a) suggest that to show evidence of mirroring, mu desynchronization studies should include both action execution and observation conditions, report EEG activity from multiple electrode sites, and evaluate attentional confounds. Firstly, we note that the current study did not include an action execution condition. This would be an interesting future avenue of research to investigate whether a comparable age-related increase in mu desynchronization is seen during execution (Marshall and Meltzoff, 2011). Secondly, we report EEG activity from multiple electrode sites, including both central and occipital sites. We note that it is unlikely that the mu desynchronization effect in our study reflects differences in attentional demands between conditions (Bazanova and Vernon, 2014). In line with Hobson and Bishop (2017a), we compared mu/alpha and beta desynchronization over central and occipital electrode sites. Although there was an indication of occipital alpha suppression, a different pattern of results emerged for central mu suppression that corresponded with the results for beta desynchronization. In addition, increasing age remained related to the action-static difference in mu desynchronization over the central sites when accounting for the difference at occipital sites.

Finally, we acknowledge the possibility that the increase in mu desynchronization in the current study could be influenced by the observation of transitive actions in the hand action condition compared to no actions in the static hand condition, or the presence of objects in five out of six hand actions compared to the absence of objects in the static hand condition. However, we do not believe that these low-level differences are driving sensorimotor effects seen here for a number of reasons. Influential findings have indicated no difference in mirror system activation for transitive versus intransitive actions (e.g., Press et al., 2008), with no moderating effect on the mu rhythm for object versus non-object directed stimuli (Fox et al., 2016). Moreover, the mirror neuron system is activated during the observation, imitation and production of both object-directed and (non-object) communicative hand gestures (Montgomery et al., 2007). The mere presence of objects does not lead to mu desynchronization (Perry and Bentin, 2009), and mu desynchronization has been shown to be greater when observing moving hands than when observing static hands, moving objects, or static objects (Pfurtscheller et al., 2007). Additionally, Papadourakis and Raos (2017) have recently shown that the mirror neurons of rhesus macaque monkeys’ respond to the observation of both transitive and intransitive actions, and these discharge differences are correlated with the kinematic differences of the actions, not with the objects’ features. This suggests that mirror neurons code the kinematics of actions and can detect subtle differences, suggesting that they have a role in encoding the goals of actions.

## 5 Conclusion

We explored the developmental trajectory of the mirror system and social cognitive processes from 10 years old through to 86 years old in a large sample of healthy individuals. We show for the first time that sensorimotor activation to action observation continues to increase throughout adulthood, with additional changes in older age. A functional mirror system is apparent from adolescence through to older age, but this is still maturing during adolescence. Moreover, an increase in sensorimotor activation to observing actions across adulthood was observed, which may reflect increasing experience with hand actions, suggesting that the mirror system continues to specialize for action observation throughout adulthood. Emotion recognition, theory of mind and empathy showed distinct developmental trajectories; these behavioral changes did not map onto alpha desynchronization elicited during action observation, although beta desynchronization during action observation was shown to be related to theory of mind ability. These distinct patterns illustrate specificity in the relationship between mirror system development and social cognitive skills.

In general, studies have largely overlooked middle-aged participants when investigating sensorimotor processes related to the mirror system and social cognitive processes, with studies either focussing on infants and children, student populations, or comparing dichotomous groups of young versus older adults. The findings of the current study highlight the importance of studying this age group, with measurable changes in both sensorimotor activation and social cognitive processes throughout adulthood. Overall, our findings indicate that the activity of the mirror system increases throughout the lifespan with measurable changes into older age that are independent from social cognitive processes.

## Author contribution

All authors contributed to study design, data collection, data analysis and interpretation, drafting the manuscript, and revising the manuscript.

## Conflicts of interest

None.

## Footnotes

1 A second multiple regression was conducted to include the quadratic and cubic terms for age due to the curvilinear relationship for the action-static difference at the central electrodes and age in the beta band. The results of this analysis were similar (R^2^ = .49, *F*(7, 293) = 40.04, *p* < 0.001), with identical predictors of the action-static difference at the central electrodes in the beta band.
