# Supplementary material for: Sensorimotor mu rhythm during action observation changes across the lifespan independently from social cognitive processes
Source: Dev Cogn Neurosci. 2019 May 17;38:100659. doi: 10.1016/j.dcn.2019.100659 (PMC6688050; doi:10.1016/j.dcn.2019.100659)
Supplement: Supplementary file 1 [file mmc1.docx]

**Supplementary Materials 1**

**Detailed Descriptions of the Social Cognition Measures**

**Reading the Mind in the Eyes Task (RMET).** Both the child and adult RMET were adapted from Baron-Cohen et al. (2001) and were used to measure emotional state recognition. EPrime software was used to present the stimuli. Participants were presented with photographs of the eye region on the center of a screen. Four possible mental state words were presented in font type Courier New and font size 28 in the top-left, top-right, bottom-left and bottom-right of the photographs. Participants were required to use a mouse to choose and click the word that best described what the person in the photograph is thinking or feeling. Each trial ended when a response was recorded (i.e., a mouse click on a word). After each trial, a blank screen appeared for 1000ms and the cursor reset to the center of the screen. Participants first completed a practice trial, after which they could ask the experimenter any questions. Participants then completed the child version (10-15 years old) with 28 test trials or the adult version (16+ years old) with 36 test trials. The presentation order of the photographs was randomised across participants. Participants’ response accuracy was recorded.

**Strange Stories.** This task was adapted from White et al. (2009) and used to measure mental state comprehension. Participants were told that they would hear some stories and that they would be asked a question at the end of each story. Eight theory of mind, eight physical and eight nature stories were audio-recorded by an adult male speaker (mean duration of clip: mental 35s, human 37s, nature 28s). EPrime software was used to present the stimuli so that the participants could read the text on the screen and hear the audio simultaneously. Participants were first presented with a blank screen for 2000ms after which the story text appeared on screen in font type Courier New and font size 30 and the audio played simultaneously. After the story was presented, a blank screen appeared for 1000ms. A question was then simultaneously presented on screen in font type Courier New and font size 30 and auditorily. Participants were required to verbally respond to the question. A microphone recorded the participants’ responses for a maximum of 60s and the participant pressed the spacebar to continue to the next story. The presentation of the stories was randomised across participants. There were 24 experimental trials with a self-directed break after a block of 8 trials. The accuracy of each response was rated 0 – 2 on a scale: 0 for an incorrect answer, 1 for a partially or implicitly correct answer, and 2 for a fully correct answer using the scoring criteria developed by White et al (2009). A ToM score was calculated from the theory of mind stories provided a ToM score (maximum = 16) and a non-ToM control score was calculated from the physical stories (maximum = 16).

Empathy Quotient. The Empathy Quotient (EQ) is a 40-item questionnaire completed either as a parent-report (10-15 years old; Auyeung, Allison, Wheelwright, & Baron-Cohen, 2012) or as a self-report measure of empathy (16+ years old; Baron-Cohen & Wheelwright, 2004). It consists of 40 statements, such as “I really enjoy caring for other people” or “My child really enjoys caring for other people” and is rated on a 4-point scale (“strongly agree”, “slightly agree”, “slightly disagree”, “strongly disagree”). The questionnaires were scored using the scoring key developed by Baron-Cohen and Wheelwright (2004) to gain a total empathy score (maximum = 80). A low score indicates low levels of empathy and a high score indicates high levels of empathy.

**Supplementary Materials 2**

Table A. Model comparisons with age as the predictor variable for each outcome variable.

| Models | | RSS | *df* | *F*Δ | *p* |
| --- | --- | --- | --- | --- | --- |
| Action-Static Difference in Alpha Power (8-13Hz) | |  |  |  |  |
|  | 1. Linear* | 181816 | 299 |  |  |
|  | 2. Quadratic | 180060 | 298 | 2.91 | .089 |
| Action-Static Difference in Beta Power (13-35Hz) | |  |  |  |  |
|  | 1. Linear | 72327 | 299 |  |  |
|  | 2. Quadratic | 69297 | 298 | 13.03 | < .001 |
|  | 3. Cubic* | 67579 | 297 | 7.55 | .006 |
|  | 4. Quartic | 67045 | 296 | 2.36 | .126 |
| RMET (Percentage Correct) | |  |  |  |  |
|  | 1. Linear | 31432 | 299 |  |  |
|  | 2. Quadratic | 29593 | 298 | 18.52 | < .001 |
|  | 3. Cubic* | 28968 | 297 | 6.41 | .012 |
|  | 4. Quartic | 28954 | 296 | 0.14 | .707 |
| Strange Stories (ToM Score) | |  |  |  |  |
|  | 1. Linear | 1444 | 299 |  |  |
|  | 2. Quadratic | 1403 | 298 | 8.68 | .003 |
|  | 3. Cubic* | 1370 | 297 | 7.17 | .008 |
|  | 4. Quartic | 1363 | 296 | 1.49 | .223 |
| Strange Stories (Control Score) | |  |  |  |  |
|  | 1. Linear | 1243 | 299 |  |  |
|  | 2. Quadratic | 1243 | 298 | 0.05 | .826 |
| Empathy Quotient (Total) | |  |  |  |  |
|  | 1. Linear* | 53203 | 299 |  |  |
|  | 2. Quadratic | 52715 | 298 | 2.76 | .098 |
| RMET = Reading the Mind in the Eyes Task, ToM = theory of mind, RSS = residual sum of squares, *F*Δ denotes the comparison of models (i.e., 1 vs. 2, 2 vs. 3, etc), * = best-fitting model | | | | | |
